# Key influences on university students’ physical activity: a systematic review using the Theoretical Domains Framework and the COM-B model of human behaviour

**DOI:** 10.1186/s12889-023-17621-4

**Published:** 2024-02-09

**Authors:** Catherine E. B. Brown, Karyn Richardson, Bengianni Halil-Pizzirani, Lou Atkins, Murat Yücel, Rebecca A. Segrave

**Affiliations:** 1 BrainPark, Turner Institute for Brain and Mental Health, Monash University, Melbourne, Australia; 2 Centre for Behaviour Change, University College London, London, UK; 3 QIMR Berghofer Medical Research Institute, Herston, Brisbane, QLD Australia

**Keywords:** University students, Physical activity, Physical exercise, Behaviour change, COM-B, Theoretical domains framework, Barriers, Facilitators

## Abstract

**Background:**

Physical activity is important for all aspects of health, yet most university students are not active enough to reap these benefits. Understanding the factors that influence physical activity in the context of behaviour change theory is valuable to inform the development of effective evidence-based interventions to increase university students’ physical activity. The current systematic review a) identified barriers and facilitators to university students’ physical activity, b) mapped these factors to the Theoretical Domains Framework (TDF) and COM-B model, and c) ranked the relative importance of TDF domains.

**Methods:**

Data synthesis included qualitative, quantitative, and mixed-methods research published between 01.01.2010—15.03.2023. Four databases (MEDLINE, PsycINFO, SPORTDiscus, and Scopus) were searched to identify publications on the barriers/facilitators to university students' physical activity. Data regarding study design and key findings (i.e., participant quotes, qualitative theme descriptions, and survey results) were extracted. Framework analysis was used to code barriers/facilitators to the TDF and COM-B model. Within each TDF domain, thematic analysis was used to group similar barriers/facilitators into descriptive theme labels. TDF domains were ranked by relative importance based on frequency, elaboration, and evidence of mixed barriers/facilitators.

**Results:**

Thirty-nine studies involving 17,771 participants met the inclusion criteria. Fifty-six barriers and facilitators mapping to twelve TDF domains and the COM-B model were identified as relevant to students’ physical activity. Three TDF domains, environmental context and resources (e.g., time constraints), social influences (e.g., exercising with others), and goals (e.g., prioritisation of physical activity) were judged to be of greatest relative importance (identified in > 50% of studies). TDF domains of lower relative importance were intentions, reinforcement, emotion, beliefs about consequences, knowledge, physical skills, beliefs about capabilities, cognitive and interpersonal skills, social/professional role and identity, and behavioural regulation. No barriers/facilitators relating to the TDF domains of memory, attention and decision process, or optimism were identified.

**Conclusions:**

The current findings provide a foundation to enhance the development of theory and evidence informed interventions to support university students’ engagement in physical activity. Interventions that include a focus on the TDF domains 'environmental context and resources,' 'social influences,' and 'goals,' hold particular promise for promoting active student lifestyles.

**Trial registration:**

Prospero ID—CRD42021242170.

**Supplementary Information:**

The online version contains supplementary material available at 10.1186/s12889-023-17621-4.

## Background

Physical activity (PA) has a powerful positive impact on all aspects of health. Regular PA can prevent and treat noncommunicable diseases [1, 2], build resilience against the development of mental illness [3], and attenuate cognitive decline [4]. Given these pervasive health benefits, increasing participation in PA is recognised as a global priority by international public health organisations. Indeed, a core aspect of the World Health Organisation’s action plan for a “healthier world” is to achieve a 15% reduction in the global prevalence of physical inactivity by 2030 [5].

Despite international efforts to reduce physical inactivity, university students frequently do not meet the recommended level of PA required to attain its health benefits. Approximately 40–50% of university students are physically inactive [6], many of whom attribute their inactivity to unique challenges associated with university life. For many students, the transition to university coincides with new academic, social, financial, and personal responsibilities [7], disrupting established routines and imposing additional barriers to the initiation or maintenance of healthy lifestyle habits such as regular PA [8]. Students’ PA tends to decline further during periods of high stress and academic pressure, such as exams and assignment deadlines [9]. This pattern has been observed across diverse university populations and cultural contexts [10–12], highlighting the importance of understanding the factors that contribute to physical inactivity among this cohort globally.

Understanding the barriers and facilitators to PA in the context of the university setting is an important step in developing effective, targeted interventions to promote active lifestyles among university students. A recently published systematic review found that lack of time, motivation, access to places to practice PA, and financial resources were primary barriers to PA for undergraduate university students [13]. A corresponding and complementary synthesis of the facilitators of PA, however, has not yet been conducted. Such a synthesis would be valuable in enabling a comprehensive understanding of the factors that influence students' PA and identifying facilitators that could be leveraged in intervention design. Furthermore, applying theoretical frameworks to understand barriers and facilitators to PA can guide the development of theory-informed, evidence-based interventions for university students that purposely and effectively target factors that influence their participation in PA.

The Theoretical Domains Framework (TDF) [14–16] and the COM-B model of behaviour [17] are two robust, gold-standard frameworks frequently used to examine the determinants of human behaviour. The TDF is an integrated framework of 14 theoretical domains (see Additional file 1 for domains, definitions, and constructs) which provide a comprehensive understanding of the key factors driving behaviour. The TDF was developed through expert consensus, synthesising 33 psychological theories (such as social cognitive theory [18, 19] and the theory of planned behaviour [20, 21] and 128 theoretical constructs (such as ‘competence’, ‘goal priority’, etc.) across disciplines identified as most relevant to the implementation of behaviour change interventions. Identifying the relative importance of theoretical domains allows intervention designers to triage which behaviour change strategies should be prioritised in intervention development [22, 23]. The TDF has been widely applied by researchers and practitioners to systematically identify which theoretical domains are most relevant for understanding health behaviour change and policy implementation across a range of contexts, including education [24], healthcare [25], and workplace environments [26].

**Fig. 1:**
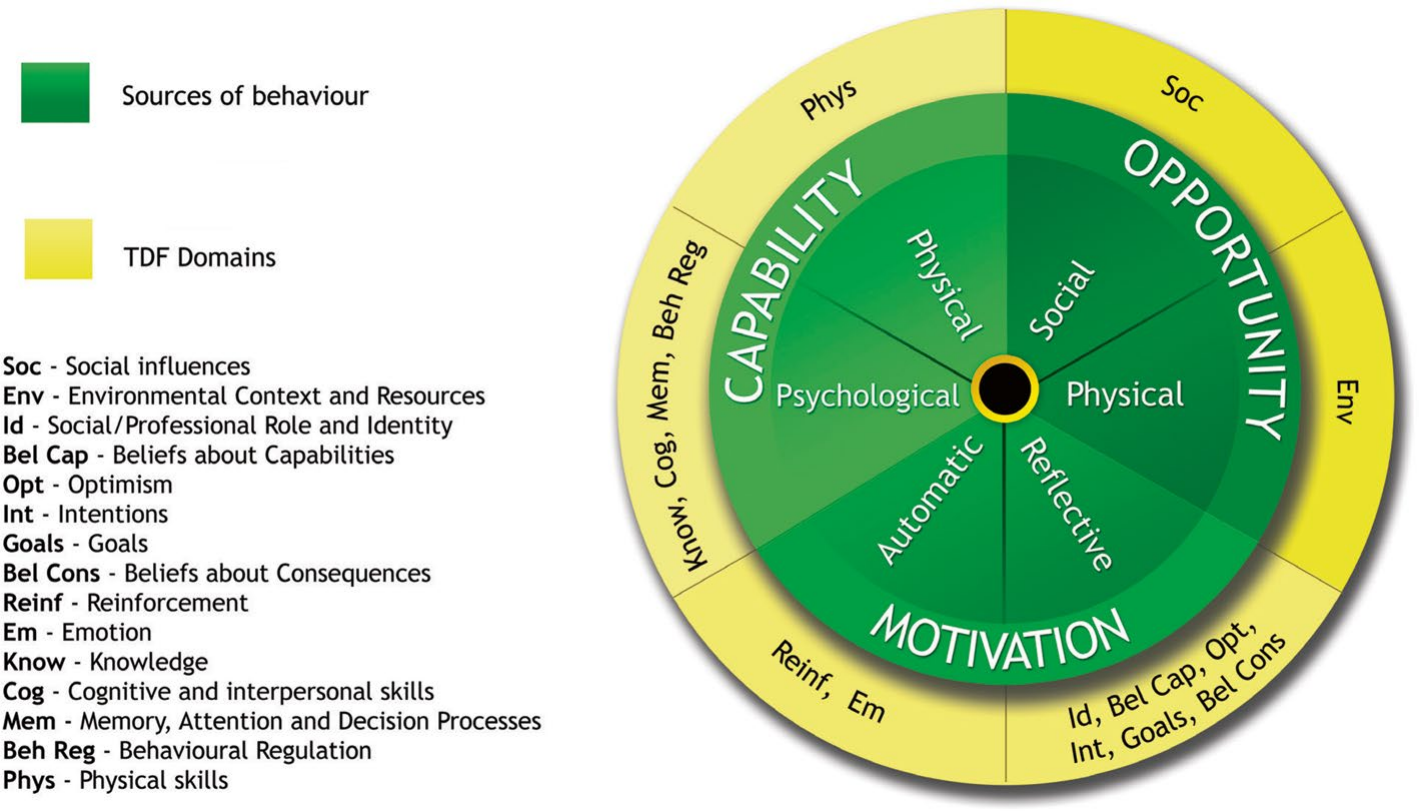
The TDF domains linked to the COM-B model subcomponents Note. Reproduced from Atkins, L., Francis, J., Islam, R., et al. (2017) A guide to using the Theoretical Domains Framework of behaviour change to investigate implementation problems. Implementation Science 12, 77. 10.1186/s13012-017-0605-9

The 14 TDF domains map onto the COM-B model (Fig. 1), which is a broader framework for understanding behaviour and provides a direct link to intervention development frameworks. The COM-B model posits that no behaviour will occur without sufficient capability, opportunity, and motivation. Where any of these are lacking, they can be strategically targeted to support increased engagement in a desired behaviour, including participation in PA. Within the COM-B model, capability can be psychological (e.g., knowledge to engage in the necessary processes) or physical (e.g., physical skills); opportunity can be social (e.g., interpersonal influences) or physical (e.g., environmental resources); and motivation can be automatic (e.g., emotional reactions, habits) or reflective (e.g., intentions, beliefs). The COM-B model was developed through a process of theoretical analysis, empirical evidence, and expert consensus as a central part of a broader framework for developing behaviour change interventions known as the Behaviour Change Wheel (BCW) [17].

Using the TDF and COM-B model to understand the barriers and facilitators to university students’ participation in PA is valuable to inform the development of effective evidence-based interventions that are tailored to address the most influential determinants of behaviour change. As such, this systematic review aimed to: a) identify barriers and facilitators to university students’ participation in PA; b) map these factors using the TDF and COM-B model; and c) determine the relative importance of each TDF domain.

## Methods

### Study design

The systematic review was conducted according to the Preferred Reporting Items for Systematic Reviews and Meta-Analyses (PRISMA) [27]. The review protocol was registered on PROSPERO (CRD42021242170).

### Search strategy

Search terms and parameters were developed in collaboration with a Monash University librarian with expertise in systematic review methodology. The following databases were searched on 15.03.2023 to identify relevant literature: MEDLINE, PsycINFO, and SPORTDiscus. Key articles were also selected for citation searching via Scopus. In consultation with a librarian, these databases were selected due to their unique scope, relevance, broad coverage, and utility. This process ensured the identified literature aligned with the aim and research topic of our systematic review. A 01.01.2010—15.03.2023 publication period was purposefully specified to account for the significant advancements in digital fitness support and tracking tools within the past decade [28], All available records were searched using the following combination of concepts in the title or abstract of the article: 1) barriers, facilitators, or intervention,^1^ 2) physical activity, 3) university, and 4) students. Each search concept was created by first developing a list of search terms relevant to each concept (e.g., for the ‘physical activity’ concept search terms included ‘physical exercise’, ‘physical fitness’, ‘sports’, ‘inactive’, ‘sedentary’, etc.). To create each concept, search terms were then searched collectively using the operator ‘OR’. Each search concept was then combined into the final search by using the operator ‘AND’. Search terms related to concepts 1, 2 and 3 included indexed terms unique and relevant to each database (i.e., Medical Subject Heading Terms for MEDLINE, Index Terms for PsycINFO, and Thesaurus terms for SPORTDiscus). The search was performed according to Boolean operators (e.g., AND, OR) (see Additional file 2 for the complete search syntax for MEDLINE). Unpublished studies were not sought.

### Selection criteria

Articles were included if they: (a) reported university students’ self-reported barriers and/or facilitators to physical activity or exercise^2^; (b) were written in English; and (c) were peer-reviewed journal articles. Articles encompassed studies directly investigating barriers and/or facilitators to students’ participation in PA and physical exercise intervention studies, where the latter reported participants’ self-reported barriers and/or facilitators to intervention adherence (see Table 1 below for full criteria).

**Table 1:** Inclusion and exclusion criteria for article selection

Inclusion criteria	Exclusion criteria
Participants: ≥ 50% of sample are university students	Participants: Not university students
Content:	
Non-intervention studies and/or physical exercise-only intervention	Intervention studies that targeted multiple health-related behaviours
Outcome:	
Specific evaluation of self-reported barrier or facilitator to PA	Preferences related to PA
	Associations or correlations with PA
Qualitative, quantitative, or mixed-methods	
Context: University	Context: Other than university
Year published: 01.01.2010—15.03.2023	
Types of publication: Peer-reviewed journal articles	
Languages: English	

Inclusion and exclusion criteria were guided by the PICOS and PICo frameworks [29]

### Study selection

Identified articles were uploaded to EndNote X9 software [30]. A duplication detection tool was used to detect duplicates, which were then screened for accuracy by CB prior to removal. The remaining articles were uploaded to Covidence to enable blind screening and conflict resolution. Articles were screened at the title and abstract level against the inclusion and exclusion criteria by author CB, and 25% were independently screened by BP. The full text of studies meeting the inclusion criteria was then screened against the same criteria by CB, and 25% were again independently screened by BP. Differences were resolved by an independent author (KR). Inter-rater agreement in screening between CB and BP was high (0.96 for title and abstract screening, 0.83 for full-text screening). The decision to dual-screen 25% of studies was strategically chosen to balance thoroughness with efficiency, ensuring both the validity of the screening criteria and the reliability of the primary screener’s decisions. This approach aligns with the protocols used in similar systematic reviews in the field (e.g., [31, 32]).

### Data extraction

Key article characteristics were extracted, including the author/s, year of publication, country of origin, participant characteristics (e.g., enrolment status, exercise engagement [if reported]), sample size, research design, methods, and analytical approach. Barriers and facilitators were also extracted for each article and subsequently coded according to the 14 domains of the TDF and six subcomponents of the COM-B model. Quantitative data were only extracted if ≥ 50% of students endorsed a factor as a barrier or facilitator. This cut-off criterion was applied to maintain focus on the most common variables of influence and aligns with other reviews synthesising common barriers and facilitators to behaviour change (e.g., [26, 33]).

A coding manual was developed to guide the process of mapping barriers and facilitators to the TDF and COM-B. All articles were independently coded by at least two authors (CB and BS, BP or KR). The first version of the manual was developed a priori, based on established guides for applying the TDF and COM-B model to investigate barriers and facilitators to behaviour [14, 34], and updated as needed via regular consultation with a co-author and TDF/COM-B designer LA to ensure the accuracy of the data extraction. Barriers and facilitators were only coded to multiple TDF domains if deemed essential to accurately contextualise the core elements of the barrier/facilitator, and when the data in individual papers was described in sufficient detail to indicate that more than one domain was relevant. For example, if ‘lack of time due to competing priorities’ was reported as a barrier to PA, this encompassed both the ‘environmental context and resources’ (i.e., time) and ‘goals’ (i.e., competing priorities) domains of the TDF. Coding conflicts were resolved via discussion with LA.

### Data analysis

The following three-step method was utilised to synthesise quantitative and qualitative data:

1. Framework analysis [35] was conducted to deductively code barriers and facilitators onto TDF domains and COM-B subcomponents. This involved identifying barriers and facilitators in each article, extracting and labelling them, and determining their relevance against the definitions of the TDF domains and COM-B subcomponents. This process involved creating tables to assist in the systematic categorisation of barriers and facilitators into relevant TDF domains and COM-B subcomponents.
2. Within each TDF domain, thematic analysis [36] was conducted to group similar barriers and facilitators together and inductively generate summary theme labels.
3. The relative importance of each TDF domain was calculated according to frequency (number of studies), elaboration (number of themes) and the identification of mixed barriers/facilitators regarding whether a theme was a barrier or facilitator within each domain (e.g., if some participants reported that receiving encouragement from their family to exercise was a facilitator, and others reported that lack of encouragement from their family to exercise was a barrier). The rank order was determined first by frequency, then elaboration, and finally by mixed barriers/facilitators.

This methodology follows previous studies using the TDF and COM-B to characterise barriers and facilitators to behaviour change and rank their relative importance [22, 23].

## Results

### Study characteristics

**Fig. 2:**
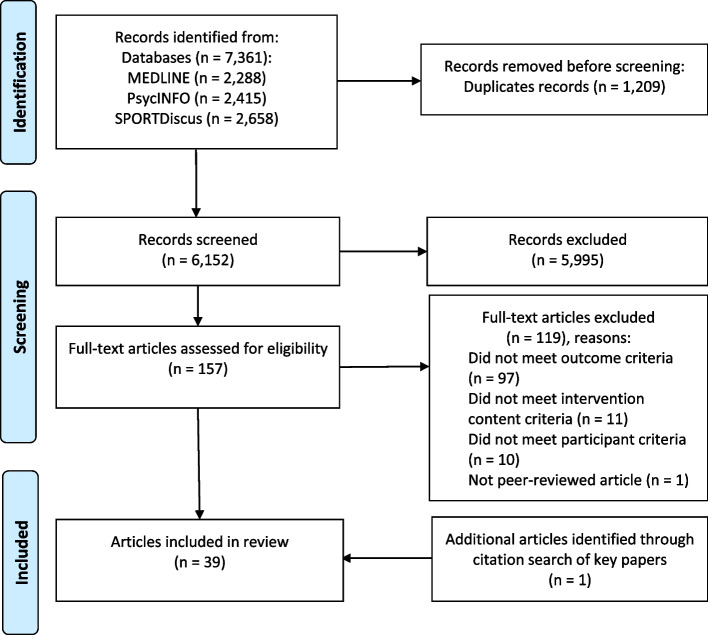
PRISMA flowchart illustrating the article selection process

Following the removal of duplicates, 6,152 articles met the search criteria and were screened based on title and abstract. A total of 5,995 articles were excluded because they did not meet the inclusion criteria (see Fig. 2 below for the PRISMA flowchart). After the title and abstract screening, 157 full-text articles were retrieved and assessed for eligibility. One additional article was identified and included following citation searching of selected key articles. Thirty-nine articles met the inclusion criteria (see Additional file 3 for a summary of these studies). Eight studies were conducted in the USA, seven in Canada, three in Germany, two each in Qatar, Spain, the United Arab Emirates, and the United Kingdom, and one each in Australia, Belgium, Columbia, Egypt, Ireland, Japan, Kuwait, Malaysia, New Zealand, Saudi Arabia, South Africa, Sri Lanka, and Uganda.

### Relative importance of TDF domains and COM-B components

Twelve of the 14 TDF domains and all six subcomponents of the COM-B model were identified as relevant to university students' PA. The rank order of relative importance of TDF domains and associated COM-B subcomponents are presented in Table 2. The three most important domains were identified in at least 54% of studies.

**Table 2:** Ranking the relative importance of each TDF domain according to the frequency of identification, thematic elaboration, and evidence of conflicting beliefs

Ranking	TDF Domain	COM-B Subcomponents	Frequency, no. of studies (*%*)	Elaboration, no. of themes	Evidence of mixed barriers/facilitators
1	Environmental context and resources	Physical opportunity	35(90)	12	Yes
2	Social influences	Social opportunity	28(72)	7	Yes
3	Goals	Reflective motivation	21(54)	3	Yes
4	Intentions	Reflective motivation	17(44)	3	Yes
5	Reinforcement	Automatic motivation	15(38)	8	Yes
6	Emotion	Automatic motivation	15(38)	2	Yes
7	Beliefs about consequences	Reflective motivation	12(31)	5	Yes
8	Knowledge	Psychological capability	11(28)	4	Yes
9	Physical skills	Physical capability	8(21)	3	Yes
10	Beliefs about capabilities	Reflective motivation	7(18)	2	Yes
11	Cognitive and interpersonal skills	Psychological capability	6(15)	1	Yes
12	Social/professional role and identity	Reflective motivation	3(8)	2	No
13	Behavioural regulation	Psychological capability	1(3)	2	No
14	Memory, attention and decision process	Psychological capability	0(0)	-	-
15	Optimism	Reflective motivation	0(0)	-	-

### Barriers and facilitators to student’s physical activity

Within the TDF domains, 56 total themes were identified, including 26 mixed barriers/facilitators, 18 facilitators and 12 barriers (Table 3). The barriers and facilitators identified within each TDF domain are summarised below (with associated COM-B subcomponent presented in parentheses), in order of relative importance:

- 1. **Environmental context and resources (Physical Opportunity)** **(*n* = 90% studies)**

**Table 3:** Summary of barriers to and facilitators of physical activity

COM-B Component	Theme	No. (%) of studies, (*n* = 39 max)	Barrier/ facilitator/ mixed	Study ID	Example excerpts and quote(s)
Capability (Physical)	TDF Domain: Skills (Physical)				
	Having the physical skills and fitness to participate in PA	5(13)	Mixed	Barrier [37–39] Facilitator [40] Mixed [41]	Barrier *Lack of skill.* [39] Facilitator *Improvement of skills.* [40]
	Lack of energy	2(5)	Barrier	[38, 42]	*“Some days my energy is low which makes it hard to get going.”* [38]
	Physical injury	2(5)	Barrier	[9, 43]	*“I had to quit basketball because of torn ligaments”* [9]
Capability (Psychological)	TDF Domain: Knowledge				
	Knowledge about the benefits of PA	5(13)	Mixed	Barrier [9] Facilitator [44–46] Mixed [47]	Barrier “*People don’t know about the benefits of physical activities*” [47] Facilitator *“If I know how the exercise activity or the exercise module can benefit me, like what I can get in return, only then am I motivated to do it”.* [45]
	Lack of knowledge about how to navigate through the gym, what exercises to do and how to use exercise equipment	4(10)	Barrier	[45, 48–50]	*“It’s a bit intimidating for one. Especially when you don’t know how to navigate through the building, like what to do or maybe how to use equipment”* [48]
	Lack of knowledge about the types of exercise programs and activities that were available on-campus, and how to sign up to participate	2(5)	Barrier	[9, 51]	*“I think some students like to try sports like hockey but they don’t know where to go”* [51]
	Knowledge about how to adapt physical activities for students with a disability to participate	2(5)	Mixed	Barrier [50] Mixed [52]	Barrier *“If they [staff] don’t know the easy ways to adapt activities and that it’s important for us to be active, then how are things ever going to change?”* [52] Facilitator *Denise [an undergraduate student with a disability] indicated she would modify the dance steps to be included.* [52]
	TDF Domain: Skills (Cognitive and interpersonal)				
	Time-management	6(15)	Mixed	Barrier [44, 53] Facilitator [47, 49, 54, 55]	Barrier *Lack of appropriate time management to include exercise into daily routine.* (Ranasinghe et al., 2016) Facilitator *“Time management had to be improved because I had more responsibilities, more of a social life and greater pressure from university work.”* [55]
	TDF Domain: Behavioural regulation				
	Self-monitoring of PA	1(3)	Facilitator	[56]	*“The important part for me is [keeping track] – I know I'm going beyond the average, like the normal number of steps for a person […]* *- it makes me more motivated.”* [56]
	Feedback on progress towards a PA-related goal	1(3)	Facilitator	[56]	*“When I […] got 80% of my goal, [I would just] go aimlessly for a walk. So that was getting me to walk more. Solely because I was on* *80% and I wanted that 100%.”* [56]
Motivation (Reflective)	TDF Domain: Social professional role and identity				
	Perceiving PA as a part of one’s self-identity	2(5)	Mixed	Barrier [50] Facilitator [38]	Barrier *“It was difficult for me to make a comeback to sport and I started in a gym that I think it was horrible, to be honest, I didn’t like it. I don’t know, maybe because I saw an aspect of sport that I didn’t contemplate in my life, in my story. Which* *was seeing sport simply for beauty purposes, you know what I mean. For the physical appearance, more superficial. So, I have always been very sensitive towards those things, you know what I mean.”* [50] Facilitator *“It is part of my identity”* [38]
	Students own professional role as a health practitioner motivates PA	1(3)	Facilitator	[44]	“*We being physiotherapists, can’t tell others to exercise without us doing them. There is a motivation from our profession to exercise.”* [44]
	TDF Domain: Beliefs about capabilities				
	Self-efficacy to participate in PA	5(13)	Mixed	Barrier [9, 44, 47, 53] Facilitator [41]	Barrier *“When I was very young I tried sports but I am not* *good at it. When we are not good at something we do not perform. So subsequently you detach from it. So after that I did not try to get involve in sports. Because I have no ability in that. I tried to improve something that I am good at.”* [44] Facilitator *The majority of students (n* = *22) felt successful and confident in their abilities to take part in their current exercise activities.* [41]
	Self-affirmation to participate in PA	2(5)	Facilitator	[37, 57]	*‘‘I’m here, I’ve gotten dressed, I’ve arrived at the gym, I’ve put in the effort, let’s start and finish this workout”* [57]
	TDF Domain: Intentions				
	Motivation to engage in PA	14(36)	Mixed	Barrier [38, 39, 41, 45, 47, 54, 58–63] Facilitator [52] Mixed [49]	Barrier *The top three perceived barriers included… lack of motivation (59.0%)* [63] Facilitator *Insistence and persistence in being physically active.* [52]
	Perception that PA is a chore	2(5)	Barrier	[38, 42]	*“See it as a task or chore you have to do to get out of the way so you can do something else.”* [38]
	Failure to follow through on intentions to engage in PA	2(5)	Barrier	[41, 53]	*“Well, I came in here thinking I would swim* *everyday or something, cause they have a number of swimming pools you know. But that just didn’t happen.”* [53]
	Self-discipline to engage in PA	2(5)	Facilitator	[9, 45]	*“You can be physically active when you have enough self-discipline to do it”* [9]
	TDF Domain: Beliefs about consequences				
	Beliefs about the physical health consequences of PA	9(23)	Mixed	Barrier [48] Facilitator [9, 37, 40, 45, 54, 57, 64, 65]	Barrier *Exercise could wait until graduation without compromising overall health.* [48] Facilitator *“You need to be active to prevent yourself from having open heart surgery”* [57]
	Maintaining or improving one’s physical appearance	5(13)	Facilitator	[37, 40, 45, 64, 65]	*The most cited motivators to exercise were improved […] physical appearance.* [65]
	Beliefs about the environmental and occupational benefits of exercise	3(8)	Facilitator	[38, 51, 57]	*“I like the speediness of my bike and not having to rely on the buses, plus it is better for the environment”* [38]
	Beliefs about the psychological benefits of exercise	3(8)	Facilitator	[37, 54, 65]	*Psychological benefits.* [54]
	Receiving advice to exercise from a credible source (e.g., health professionals)	2(5)	Facilitator	[54, 66]	*Being physically active for health is not a concern unless prescribed by the doctor.* [54]
	TDF Domain: Goals				
	Prioritisation of PA compared to other activities	20(51)	Mixed	Barrier [9, 37, 38, 40, 41, 43–45, 49, 53, 54, 57–60, 62, 63, 65] Mixed [42, 48]	Barrier *Being students, academic performance had become their priority and it had outweighed the* *perceived health benefits from exercise.* [45] Facilitator *[Participants who frequently exercised] saw exercise as something that needed to be prioritised.* [48]
	Engaging in PA to achieve an external goal	4(10)	Facilitator	[41, 54, 57, 63]	*Students chose to engage in activities because they deemed them valuable and a necessary component of achieving an external goal* [41]
	Setting specific PA-related goals	1(3)	Facilitator	[56]	*Many participants expressed that they benefited from goal setting. They believed that setting a goal… kept them accountable for their physical activity performance and motivated them to reach that goal* [56]
Motivation (Automatic)	TDF Domain: Reinforcement				
	Experiencing the positive benefits of PA	9(23)	Facilitator	Facilitator [9, 37, 38, 45, 47–49, 51, 63]	Facilitator *“I love the therapeutic benefits of going on a run when I am stressed.”* [38]
	Past and current habits and routines	7(18)	Mixed	Barrier [58] Facilitator [9, 37, 38, 45, 48, 54]	Barrier *“I prefer doing things regularly and somehow the hobby, the physical activity, it’s suffering from this [inflexible university schedule]”* [58] Facilitator *In various ways, participants articulated the idea that developing an exercise habit at a young age renders benefits throughout life* [48]
	Experiencing discomfort during or after PA	3(8)	Barrier	[38, 45, 67]	*“The thing about exercise that I don’t like is that after exercise, especially when the body is just beginning to adapt to exercising, I’ll get body aches and pain after”.*[45]
	Sense of accomplishment in relation to PA	2(5)	Mixed	Barrier [38] Facilitator [41]	Barrier *“[Running] never puts me outside my comfort zone or challenges me…”* [38] Facilitator *Over half (n* = *15) cited feeling a sense of accomplishment when they exercised* [41]
	Receiving positive feedback from others	1(3)	Facilitator	[57]	*Additionally, when compliments and positive feedback were given, although not inherently persuasive in nature, students felt these messages increased their efficacy and encouraged them to continue to be physically active.* [57]
	Receiving incentives	1(3)	Facilitator	[47]	*“Making offers will help—if they do many exercise then they have to pay less to use the gym”* [47]
	Experiencing a sense of achievement	1(3)	Facilitator	[40]	*It was found that fun and enjoyment in physical activity/sport is associated primarily with… goal achievement/winning* [40]
	TDF Domain: Emotion				
	Enjoyment	10(26)	Mixed	Barrier [9, 47, 58] Facilitator [37, 51, 54, 64, 66] Mixed [38, 45]	Barrier *“I don’t like any kind of sport”* [9] Facilitator *“Getting the heart rate up is always enjoyable.”* [38]
	Poor mental health and negative affectivity (e.g., fear, sadness, self-consciousness, stress)	8(21)	Barrier	[39, 45–48, 57–59]	*“My mood definitely impacts my activity behavior. Like if I’m feeling down or like I don’t know it’s harder for me to get to the gym and I know it’s better for me to go to the gym, like I always know I’m going to feel better, but sometimes I just don’t* *feel like it or I’m super down or I don’t know and then I just even feel like doing anything”* [57]
Opportunity (Physical)	TDF Domain: Environmental context and resources				
	Lack of time	25(64)	Barrier	[8, 9, 37, 38, 40, 41, 43, 45–49, 53–55, 59–62, 65, 68–72]	*“I became aware of the barriers … where you’ll* *be studying at night time and you wouldn’t* *have time to do any exercise and you’re* *working all day, so …”* [70]
	Easily accessible exercise options, facilities and equipment	24(62)	Mixed	Barrier [37, 39, 43, 44, 48, 53, 58, 59, 67] Facilitator [9, 41, 49, 54, 72] Mixed [38, 45–47, 50–52, 60, 65, 66]	Barrier *“I know some people did not do it (exercise program), because they would have to go all the way down to lower residence to take the class … they wanted to join but it was a pain to go down (to lower residence) …”* [43] Facilitator “*Here on this university campus all sports facilities are close to one another and therefore it ‘invites’ to be physically active”* [9]
	Financial costs	10(26)	Mixed	Barrier [38, 39, 47, 49, 59, 62] Facilitator [37, 50, 55] Mixed [9]	Barrier *“In all kinds of sports price is often a barrier to participate…”* [9] Facilitator *Furthermore, free student recreation center memberships and intramural sports attracted increased participation due to financial pressures, such as the cost of equipment, and the expense participating in club sports.* [55]
	Weather appropriate for PA	7(18)	Mixed	Barrier [9, 47, 50, 54, 58, 71] Facilitator [51]	Barrier *The climate is not suitable for practising exercise.* [71] Facilitator *“We went snowboarding and skating. I like these two winter sports, because in Chine, in my city, there is hardly any snow in winter so there is no chance to do that.”* [51]
	Safe and enjoyable environment	6(15)	Mixed	Barrier [59, 60] Facilitator [58, 72] Mixed [38, 50]	Barrier *Lack of safe sporting places.* [60] Facilitator *…safe surroundings that encouraged walking recreationally* [72]
	Incidental PA (at home, work or university)	5(13)	Mixed	Barrier [72] Facilitator [54, 59, 66] Mixed [73]	Barrier *Students employed in sedentary jobs (e.g., administrative assistant, tutor) perceived their job had a negative impact on their physical activity.* [72] Facilitator *Including PA into commute/going to places (walk to school, take stairs)* [54]
	Access to a variety of physical activities	4(10)	Mixed	Barrier [58] Facilitator [37, 49, 51]	Barrier *Others named that the kind of sport they were interested in, was not offered by the university sports programme* [58] Facilitator *“The program offers every kind of activity imaginable, from climbing to kayaking and even field trips”* [37]
	Information provision regarding on-campus exercise options	4(10)	Mixed	Barrier [53, 58, 73] Facilitator [50]	Barrier *“It’s not like we get a lot of information about* *sports teams and stuff… the [athletic centre] website is so stupid! Even when you try and find out the information, you get sent to this link and that, you get a schedule… it’s like why am I wasting my time? Had I had more info, I would have been more motivated.”* [53] Facilitator *Other participants went further to add that their associations provide information on PA opportunities*. [50]
	Lack of personalised physical activities to cater to individual fitness needs	3(8)	Barrier	[37, 53, 60]	Barrier *Some students expressed the need for more beginner courses to accommodate first-time participants* [37]
	Lack of university policy and promotion to encourage PA	3(8)	Barrier	[9, 44, 73]	*The Institute (university curriculum and structure) has not promoted or motivated them to do sports or physical activity.* [44]
	Health-concerning behaviours associated with university	2(5)	Barrier	[9, 57]	*A second key environmental component that could serve as a barrier to activity was the college scene itself. Students recognized how habits such as eating poorly, drinking, and physical inactivity increased during college.* [57]
	Listening to music while exercising	1(3)	Facilitator	[38]	*Listening to music while exercising.”* [38]
Opportunity (Social)	TDF Domain: Social influences				
	Exercising with others (impacts accountability, support, enjoyment, friendships etc.)	25(64)	Mixed	Barrier [38, 39, 44, 48, 53, 54, 56, 58] Facilitator [37, 40, 43, 46, 47, 50, 52, 55, 57, 66, 72] Mixed [9, 45, 49, 51, 63, 64]	Barrier *Infrequent exercisers described how the absence of having friends to exercise with was a real barrier.* [48] Facilitator *Having an exercise partner and having a friend to exercise with were found as important cues to action in this study.* [63]
	Encouragement from others to be physically active (e.g., friends, family, teachers etc.)	12(31)	Mixed	Barrier [44, 47, 52, 60] Facilitator [45, 47, 50] Mixed [9, 41, 48, 53, 66]	Barrier *“Most of my teachers, parents and others motivated to get education and go to the university. So most our target was to go to a university and get a degree which will help to get a job and settle in life. Teachers told us ‘Don’t do sports! Concentrate on education!’ They did not encouraged us to do sports”* [44] Facilitator *“Well, my dad is pretty pro-working out and staying fit. I think he started a few years ago, and noticed the benefits. So he wants me to do some bodybuilding and stuff… he is like anything you want to take, just take it and I’ll go pay for it.”* [53]
	Competition or relative comparison to others	7(18)	Mixed	Facilitator [9, 37, 38, 64] Mixed [50, 56, 57]	Barrier *Some participants mentioned that comparison to higher standards could be rather demotivating and confronting, especially when they failed to achieve as many steps as others.* [56] Facilitator *In the exercise group…both men and women were more likely to be motivated to exercise for enhancement of competitiveness* [64]
	Sociocultural norms and religion	6(15)	Mixed	Barrier [44, 47, 48] Mixed [51, 54, 66]	Barrier *… strict rules ‘in terms of religion and culture’ were imposed from her upbringing, impeding her use of the campus athletic facility. Another woman explained that ‘culture does influence whether you exercise’ in describing a sharp contrast between her cultural upbringing and the norms inside a college campus athletic facility.* [48] Facilitator *PA is an important part of the Japanese* *culture.* [54]
	Gender	6(15)	Mixed	Barrier [44, 47–49, 59] Mixed [66]	Barrier *“I don’t feel comfortable sometimes because it’s filled with guys and they just stare and look* *at you, and I don’t feel comfortable when people watch me work out”* [48] Facilitator *…culture was seen as a facilitator for* *physical activity from the males’ perspectives.* [66]
	Being stared at while engaging in PA	4(10)	Barrier	[45, 48–50]	*The experience of being stared at by others can have negative effects on people with disabilities.* [50]
	Exercise role models (positive and negative)	3(8)	Facilitator	[9, 47, 57]	*‘‘That person doesn’t work out; I don’t want to become that.”* [57]

Excerpts from authors interpretations of barriers and facilitators to exercise are italicised without quotation marks. Direct participant quotes are italicised with quotation marks. *PA* Physical activity

The most frequent barrier to PA across all TDF domains was ‘lack of time’, most often in the context of study demands. Time constraints were exacerbated by long commutes to university, family responsibilities, involvement in co-curricular activities, and employment commitments. Students’ need for ‘easily accessible exercise options, facilities and equipment’ was a recurring theme. PA was deemed inaccessible if exercise facilities and other infrastructure to support PA, such as bike paths and running trails, were situated too far from the university campus or students’ residences, or if fitness classes were scheduled at inconvenient times. ‘Financial costs’ emerged as a theme. The costs associated with accessing exercise facilities, equipment and programs consistently deterred students from engaging in PA. The desire for ‘safe and enjoyable’, ‘weather appropriate’ environments for PA were frequently reported. Participating in outdoor PA in green spaces or near water increased enjoyment, provided the environment felt safe and weather conditions were suitable for PA. Factors related to students’ home, work, and university environment impacted their participation in ‘incidental PA’. Incidental PA was influenced by whether students engaged in domestic house chores, and manual work, and actively commuted to university and between classes on-campus. Students’ ‘access to a variety of physical activities’ and ‘information provision regarding on-campus exercise options’ impacted their PA. Students most often had access to a wide variety of physical activities, however, it could be difficult to access information about what types of activities were available on-campus and how to sign up to participate. The ‘lack of personalised physical activities to cater to individual fitness needs’ was a barrier, particularly for students with low levels of PA who required beginner-oriented programs. Another barrier was the ‘lack of university policy and promotion to encourage PA’, which led students to perceive that there was no obligation to participate in PA and that the university did not value it. ‘Health-concerning behaviours associated with university’, including poor diet, increased alcohol intake and sedentary behaviour, negatively impacted students’ PA. ‘Listening to music while exercising’ was a facilitator.

- 2. **Social influences (Social Opportunity) (*n* = 72% studies)**

Within social influences, ‘exercising with others’ emerged as the most frequent theme. Doing so increased students’ accountability, enjoyment and motivation, and helped them to overcome feelings of intimidation when exercising alone. Having a lack of friends to exercise with was a particular concern for students who were new to exercise or infrequently participated in PA. Receiving ‘encouragement from others to be physically active’, such as family members, friends, peers, and fitness instructors, shaped students’ values toward PA and enhanced their motivation and self-efficacy. Students’ family members, friends and teachers discouraged PA if it was not valued, or in favour of other priorities, such as academic commitments. Another recurrent theme was ‘competition or relative comparison to others’. While most students were motivated by competition, a minority felt demotivated if they compared themselves to others with higher PA standards, especially if they failed to achieve similar PA goals. Sociocultural norms influenced barriers/facilitators to PA across different cultures, and between various groups, such as international versus domestic students, and women versus men. Students from Japan and Hawaii viewed PA as an important part of their culture, in contrast to students from the Philippines who described the opposite. Participation in PA enabled international students to integrate with domestic students and learn about the local culture, however cultural segregation was a barrier to participation in university team sports. For female students from some middle-eastern countries, including Saudi Arabia, the UAE and Qatar, cultural norms made it impermissible for women to engage in PA, particularly compared to men. Religion also differentially impacted barriers/facilitators between women and men. Muslim women reported that Islamic practices, such as needing to engage in PA separately from men, be accompanied by a male family member while going outdoors, or dress modestly, posed additional barriers to PA. However, one study reported that Islamic teachings generally encouraged PA for both women and men by emphasising the importance of maintaining good health. Other gender-specific barriers were identified. Women often felt unwelcome or intimidated by men in exercise facilities, partly due to the perception that these facilities were tailored toward “masculine” sports and/or dominated by men. ‘Being stared at while engaging in PA’ was another barrier, impacting both women and students with a disability. A less common facilitator was the influence of both positive and negative ‘exercise role models’. For example, students practiced PA because they aspired to be like someone who was physically active, or because they did not want to be like someone who was not physically active.

- 3. **Goals (Reflective Motivation) (*n* = 54%)**

‘Prioritisation of PA compared to other activities’ was the most common theme within goals. Students frequently prioritised other activities, such as study, social activities, or work, over PA. However, those who played team sports or regularly practiced PA were more inclined to prioritise it for its recognised health benefits (i.e., stress management), and its role in enhancing confidence. Additional facilitators included ‘engaging in PA to achieve an external goal’, such as improving one’s appearance, and ‘setting specific PA-related goals’ as a means to enhance accountability.

- 4. **Intentions (Reflective Motivation) (*n* = 44%)**

Within intentions, ‘motivation to engage in PA’ was the most common theme. Students most often noted a lack of self-motivation for PA. Less frequent barriers included perceiving PA as an obligatory or necessary "chore", and ‘failing to follow through on intentions to engage in PA’. Conversely, ‘self-discipline to engage in PA’ emerged as a facilitator that assisted students in maintaining a regular PA routine.

- 5. **Reinforcement (Automatic Motivation) (*n* = 38%)**

The most frequent facilitator within reinforcement was ‘experiencing the positive effects of PA’ on their health and wellbeing. These included physical health benefits (i.e., maintaining fitness), psychological benefits (i.e., stress reduction), and cognitive health benefits (i.e., enhanced academic performance). Conversely, barriers arose from ‘experiencing discomfort during or after PA’ due to pain, muscle soreness or fatigue. ‘Past and current habits and routines’ was a theme. Students were more likely to participate in PA if they had established regular exercise routines, and that forming these habits at an early age made it easier to maintain them later in life. However, maintaining a regular PA routine was difficult in the context of inflexible university schedules. Students’ ‘sense of accomplishment in relation to PA’ was a theme. Students were less likely to feel a sense of accomplishment after participating in PA if it was not physically challenging. Consistent facilitators were ‘receiving positive feedback from others’ after engaging in PA, such as compliments, and ‘receiving incentives’, such as reducing the cost of gym memberships if students participated in more PA. ‘Experiencing a sense of achievement’ after reaching a PA-related goal or winning a sports match also served as a facilitator.

- 6. **Emotion (Automatic Motivation) (*n* = 38%)**

‘Enjoyment’ was the most frequently cited emotional theme. Most students reported that PA was fun and/or associated with positive feelings, however, a minority described PA as unenjoyable, boring, and repetitive. Students’ ‘poor mental health and negative affectivity’ (such as feeling sad, stressed or self-conscious, as well as fear of injury and pain), adversely impacted their motivation to be physically active.

- 7. **Beliefs about consequences (Reflective Motivation) (*n* = 31%)**

‘Beliefs about the physical health consequences of PA’ was the most recurrent barrier/facilitator. Most students understood that PA was essential for maintaining good health and preventing illness. However, some students who rarely or never engaged in PA believed they could delay pursuing an active lifestyle until they were older without compromising their health. Participating in PA to ‘maintain or improve one’s physical appearance’ acted as a facilitator. This motivation was most often cited in contexts such as increasing or decreasing weight, changing body shape or enhancing muscle tone. Beliefs about the positive environmental, occupational and psychological impacts of PA also served as facilitators. Students were motivated to participate in PA due to the environmental benefits of using active transport. They also acknowledged the importance of being physically fit for work and believed that being active was beneficial for mental health. ‘Receiving advice to participate in PA from a credible source’, such as a health professional, further facilitated students’ motivation to be active.

- 8. **Knowledge (Psychological Capability) (*n* = 28%)**

'Knowledge about the benefits of PA’, encompassing an understanding of the various types of benefits (i.e., physical, mental, or cognitive) and the biological mechanisms by which PA brings about these changes was identified as the most common knowledge theme. Being aware of these benefits positively influenced students’ motivation to be physically active. Conversely, students’ lack of knowledge about the gym environment and the programs available were barriers to PA. Regarding the gym environment, students’ ‘lack of knowledge about how to navigate through the gym, what exercises to do, and how to use exercise equipment’ amplified feelings of intimidation. Likewise, ‘lack of knowledge about the types of exercise programs and activities that were available on-campus, and how to sign up to participate’ were all barriers. A unique theme emerged concerning ‘knowledge about how to adapt physical activities for students with a disability’. Students with a disability described how fitness instructors often had a limited understanding of how to modify activities to enable them to participate. However, students with a disability were able to overcome this barrier if they possessed their own knowledge about how to tailor physical activities to meet their specific needs.

- 9. **Physical skills (Physical Capability) (*n* = 21%)**

The most prevalent theme within physical skills was ‘having the physical skills and fitness to participate in PA’. A lack of physical skills was most frequently a hindrance to PA. Additional obstacles to PA included being physically inhibited due to a ‘lack of energy’ or ‘physical injury’.

- 10. **Beliefs about capabilities (Reflective Motivation) (*n* = 18%)**

Within beliefs about capabilities, ‘self-efficacy to participate in PA’ was the most recurrent theme. Students who doubted their success in becoming physically active or who lacked confidence in their ability to initiate PA or participate in sport were less motivated to take part. A less frequent facilitator was students’ ‘self-affirmation to participate in PA’, often referring to positive cognitions about one’s own physical abilities.

- 11. **Cognitive and interpersonal skills (Psychological Capability) (*n* = 15%)**

‘Time-management’ was the only theme identified within cognitive and interpersonal skills. Students who struggled to manage their time effectively found it difficult to incorporate regular PA into their daily routine.

- 12. **Social/professional role and identity (Reflective Motivation) (*n* = 8%)**

The most frequent theme within social/professional role and identity was ‘perceiving PA as a part of one’s self-identity’. Students who engaged regularly in PA often considered it integral to their identity. Conversely, students who perceived they did not align with the aesthetic and superficial stereotypes commonly associated with the fitness industry felt less motivated to be active. A specific facilitator emerged among physiotherapy students, who were motivated to be active due to the emphasis on PA within their profession.

- 13. **Behavioural regulation (Psychological Capability) (*n* = 3%)**

Within the domain of behavioural regulation, two facilitators were equally prevalent: ‘self-monitoring of PA’ and ‘feedback on progress towards a PA-related goal’. By keeping track of their step count and receiving feedback on walking goals, students were motivated to exceed the average number of daily steps or achieve their personal PA targets.

- 14. **Memory, attention, and decision process (Psychological Capability); Optimism (Reflective Motivation) (*n* = 0%)**

No barriers or facilitators relating to the TDF domains of memory, attention and decision process, or optimism were identified.

## Discussion

This systematic review used the TDF and COM-B model to identify barriers and facilitators to PA among university students and rank the relative importance of each TDF domain. It is the first review to apply these frameworks in the context of increasing university students’ participation in PA. Twelve TDF domains across all six sub-components of the COM-B model were identified. The three most important TDF domains were ‘environmental context and resources’, ‘social influences’, and ‘goals’. The most common barriers and facilitators were ‘lack of time’, ‘easily accessible exercise options, facilities and equipment’, ‘exercising with others’, and ‘prioritisation of PA compared to other activities’.

The most common barrier to PA was perceived lack of time. This is consistent with previous findings among university students [13, 74] and across other populations [24], For students, lack of time was frequently attributed to a combination of competing priorities and underdeveloped time management skills. Students predominantly prioritised study over PA, as performing well at university is a valued goal and there is a common perception that spending time exercising (at the expense of study) will impede their academic success [53, 58]. Evidence from cognitive neuroscience research, however, suggests that this is a mistaken belief. In addition to its broad physical and mental health benefits, a growing body of evidence demonstrates regular PA can change the structure and function of the brain.

These changes can, in turn, enhance numerous aspects of cognition, including memory, attention, and processing speed [4, 75–77], and buffer the negative impact of stress on cognition [78], all of which are important for academic success. However, students are typically unaware of the brain and cognitive health benefits of PA and its potential to improve academic performance, particularly compared to the physical health benefits [37, 40, 64]. Interventions that position participating in PA as a conduit for helping, rather than hindering, academic goals could increase the relative importance of PA to students and therefore increase their motivation to regularly engage in it. The impact that interventions of this nature have on students’ PA is yet to be empirically assessed.

Ineffective time management also contributed to students’ perceived lack of time for PA. Students reported tendencies to procrastinate in the face of overwhelming academic workloads, which left limited time for PA [53]. Additionally, students lacked an understanding of how to organise time for PA around academic timetables, social and family responsibilities, co-curricular activities, and employment commitments [9, 44, 53, 59]. To address these challenges, efforts to develop students’ time management skills will be useful for enabling students to regularly participate in PA. Goal-setting and action planning are two specific examples of such skills that can be integrated into interventions to help students initiate and maintain a PA routine [79]. For example, goal-setting could involve setting a daily PA goal, and action planning could involve planning to engage in a particular PA at a particular time on certain days.

While the most common determinants of university students’ PA levels were not influenced by specific demographic characteristics, several barriers disproportionately impacted women and students with a disability. These findings are in keeping with evidence that PA is lower among these equity-deserving groups compared with the general population [68, 80]. For women, particularly those from Middle Eastern cultures, restrictions were often tied to religious practices and sociocultural norms that limited their opportunities to engage in PA [45, 48, 66]. Additionally, a substantial number of women felt intimidated or self-conscious when exercising in front of others, especially men [48, 49]. They also felt that exercise facilities were more often tailored towards the needs of men, leading to a perception that they were unwelcome in exercise communities [45, 48]. Consequently, women expressed a desire for women-only spaces to exercise to help them overcome these gender-specific barriers to PA [47, 48, 66]. Furthermore, students with a disability faced physical accessibility barriers and perceived stigmatisation that deterred them from PA [50, 52]. The lack of accessible exercise facilities and suitable equipment, programs, and education regarding how to adapt physical activities to accommodate their needs limited their opportunity and ability to participate [52]. Moreover, students with a disability felt stigmatised by others for not fitting into public perceptions of ‘normality’ or the aesthetic values and beauty standards often portrayed by the fitness industry [50]. These barriers for both equity-deserving groups of students are deeply rooted in historical stereotypes that have traditionally excluded women and people with a disability from engaging in various types of PA [81, 82]. Despite growing awareness of these issues, PA inequalities persist due to narrow sociocultural norms, and a lack of diverse representation and inclusion in the fitness industry and associated marketing campaigns [83, 84]. A concerted effort to address PA inequalities across the university sector and fitness industry more broadly is needed. One approach for achieving this is to develop interventions that are tailored to the unique needs of equity-deserving groups, emphasise inclusivity, diversity, and empowerment, and feature women and people with a disability being active.

The “This Girl Can” [85] and “Everyone Can” [86] multimedia campaigns are two examples of health behaviour interventions that were co-developed with key stakeholders (i.e., women and people with a disability, respectively) to tackle PA inequalities. The “This Girl Can” campaign has reached over 3 million women and girls, projecting inclusive and positive messages that aim to empower them to be physically active. Following the widespread reach of the “This Girl Can” campaign, the “Everybody Can” campaign was launched to support the inclusion of people with a disability in the PA sector. Although not tailored for university students, these campaigns provide a useful example for developing interventions that are specifically designed to address key barriers preventing women and people with a disability from participating in PA.

Across the tertiary education sector globally, efforts to elevate opportunities and motivation to include PA as a core part of the student experience will be beneficial for promoting students’ PA at scale. Two intervention approaches that can be implemented to facilitate such an endeavour are environmental restructuring and enablement [17]. These intervention approaches should involve the provision of accessible low-cost exercise options, facilities, and programs, integrating PA into the university curriculum, and mobilising student and staff leadership to encourage students’ participation in PA [9]. Although there is evidence that these approaches can be effective in promoting sustained PA throughout students’ university years and beyond [87], implementation measures such as these are complex. Implementation requires aligning student activity levels with broader university goals and is further complicated by having to compete with other funding priorities and resource allocations. Notably, due to the negative impact of the COVID-19 pandemic on university students’ physical and mental health [88, 89], the post-pandemic era has seen many universities prioritise enhancing student health and wellbeing alongside more traditional strategic goals like academic excellence and workforce readiness. Despite the potential for PA to be used as a vehicle for supporting these strategic goals there is an absence of data on the extent to which this is occurring in the university sector. The limited evidence in this area suggests that some universities have made efforts to support students’ mental health by referring students who access on-campus counselling services to PA programs [90]. However, the uptake and efficacy of such initiatives is rarely assessed, and even less is known about whether PA is being used to support other strategic goals, such as academic success. Therefore, while the potential is there for the university sector to use PA to support students’ mental health and academic performance, to be successful this needs to become a strategic university priority. Given that these strategic priorities are set at the senior leadership level, engaging senior university staff in intervention design and promotion efforts is important to enhance the value of PA in the tertiary education sector.

### Implications for intervention development

The current findings provide a high-level synthesis of the most common barriers and facilitators to university students’ physical activity. These findings can be leveraged with behavioural intervention development tools and frameworks (e.g., the BCW [17], Obesity-Related Behavioural Intervention Trials model [91], Intervention Mapping [92], and the Medical Research Council guidelines for developing complex interventions [93, 94]) to develop evidence-based interventions and policies to promote PA. Given that the TDF and COM-B model are directly linked to the BCW framework, applying this process may be particularly useful to translate the current findings into an intervention.

Additionally, current findings can be triangulated with data directly collected from key stakeholders to assist in the development of context-specific interventions. Best practice principles for developing behavioural interventions recommend this approach to ensure a deep understanding of the barriers and facilitators that need to be targeted to increase the likelihood of behaviour change [17]. Consulting stakeholders directly (i.e., university students and staff) to understand their perspectives on the barriers and facilitators to students’ PA also enables an intervention to be appropriately tailored to the target population’s needs and implementation setting. Studies continue to demonstrate the effectiveness of this approach, especially when framed within the context of frameworks directly linked to intervention development frameworks, such as the TDF [95].

### Strengths and limitations

The findings of this review should be considered with respect to its methodological strengths and limitations. The credibility and reliability of the research findings are supported by a systematic approach to screening and analysing the empirical data, along with the use of gold-standard behavioural science frameworks to classify barriers and facilitators to PA. The inclusion of qualitative, quantitative, and mixed-methods studies of both barriers and facilitators to students’ PA allowed for a comprehensive understanding of the factors that influence students’ PA that have not previously been captured.

While the present review elucidates students’ own perspectives of the factors that influence their activity levels, other stakeholders such as university staff, will also influence the adoption, operationalisation, and scale of PA interventions in a university setting. It will be important for future research to explore factors that influence university decision-makers in these roles to inform large-scale strategies for promoting students' PA.

Additionally, only one study included in the review used the TDF to explore barriers and facilitators to PA [47]. Therefore, it is possible that certain TDF domains may not have been identified because students were not asked relevant questions to assess the influence of those domains on their PA. For instance, domains such as ‘memory, attention, and decision process’, and ‘optimism’ are likely to play a role in understanding the barriers and facilitators to PA despite not being identified in this review.

Moreover, quantitative data were only extracted if ≥ 50% of students endorsed the factor as a barrier or facilitator to PA. This threshold was purposefully applied to maintain a focus on the TDF domains most universally relevant to the broad student population in the context of understanding their barriers and facilitators to PA. It is possible that less frequently reported barriers and facilitators, which may not be as prominently featured in the results, could be relevant to specific groups of students, such as those identified as equity-deserving.

Lastly, a quality appraisal of the included studies was not undertaken. This decision was informed by the aim of the review, which was to describe and synthesise the literature to subsequently map data to the TDF and COM-B rather than assess the effectiveness of interventions or determine the strength of evidence. However, this decision, combined with dual screening 25% of the studies and excluding unpublished studies and grey literature, may introduce sources of error and bias, which should be considered when interpreting the results presented.

## Conclusion

PA is an effective, scalable, and empowering means of enhancing physical, mental, and cognitive health. This approach could help students reach their academic potential and cope with the many stressors that accompany student life, in addition to setting a strong foundation for healthy exercise habits for a lifetime. As such, understanding the barriers and facilitators to an active student lifestyle is beneficial. This systematic review applied the TDF and COM-B model to identify and map students’ barriers and facilitators to PA and, in doing so, provides a pragmatic, theory-informed, and evidence-based foundation for designing future context-specific PA interventions. The findings from this review highlight the importance of developing PA interventions that focus on the TDF domains ‘environmental context and resources’, ‘social influences’, and ‘goals’, for which intervention approaches could involve environmental restructuring, education, and enablement. If successful, such strategies could make a significant contribution to improving the overall health and academic performance of university students.

### Supplementary Information

**Additional file 1. **Theoretical Domains Framework domains, definitions, and constructs.

**Additional file 2. **Search syntax for Ovid MEDLINE.

**Additional file 3. **Summary of study characteristics.

**Additional file 4. **PRISMA Checklist.

## Data Availability

The review protocol is available on PROSPERO. The datasets used and/or analysed during the current study and materials used are available from the corresponding author on reasonable request.

## Abbreviations

BCW: Behaviour Change Wheel
COM-B: Capability, Opportunity, Model-Behaviour
PA: Physical activity
PRISMA: Preferred Reporting Items for Systematic Reviews and Meta-Analyses
PROSPERO: International Prospective Register of Systematic Reviews
TDF: Theoretical Domains Framework

## Footnotes

1 The term ‘intervention’ was included to identify student barriers and facilitators to engaging in implemented physical activity interventions.

2 Physical exercise is defined as “a subset of physical activity that is planned, structured, and repetitive”, and purposefully focused on the improvement or maintenance of physical fitness, whereas physical activity is defined as “any bodily movement produced by skeletal muscles that results in energy expenditure” [96].
