# Supplementary material for: Key influences on university students’ physical activity: a systematic review using the Theoretical Domains Framework and the COM-B model of human behaviour
Source: BMC Public Health. 2024 Feb 9;24:418. doi: 10.1186/s12889-023-17621-4 (PMC10854129; doi:10.1186/s12889-023-17621-4)
Supplement: Supplementary file 1 — Additional file 1. Theoretical Domains Framework domains, definitions, and constructs. [file 12889_2023_17621_MOESM1_ESM.docx]

| **Additional file 1.** Theoretical Domains Framework domains, definitions, and constructs (reproduced from Atkins et al., 2017). | | |
| --- | --- | --- |
| Theoretical domain | Definition | Constructs |
| Knowledge | An awareness of the existence of something | Knowledge (including knowledge of condition/scientific rationale)  Procedural knowledge  Knowledge of task environment |
| Skills | An ability or proficiency acquired through practice | Skills  Skills development  Competence  Ability  Interpersonal skills  Practice  Skill assessment |
| Social/professional role and identity | A coherent set of behaviours and displayed personal qualities of an individual in a social or work setting | Professional identity  Professional role  Social identity  Identity  Professional boundaries  Professional confidence  Group identity  Leadership  Organisational commitment |
| Beliefs about capabilities | Acceptance of the truth, reality or validity about an ability, talent or facility that a person can put to constructive use | Self-confidence  Perceived competence  Self-efficacy  Perceived behavioural control  Beliefs  Self-esteem  Empowerment  Professional confidence |
| Optimism | The confidence that things will happen for the best or that desired goals will be attained | Optimism  Pessimism  Unrealistic optimism  Identity |
| Beliefs about consequences | Acceptance of the truth, reality, or validity about outcomes of a behaviour in a given situation | Beliefs  Outcome expectancies  Characteristics of outcome expectancies  Anticipated regret  Consequents |
| Reinforcement | Increasing the probability of a response by arranging a dependent relationship, or contingency, between the response and a given stimulus | Rewards (proximal/distal, valued/not valued, probable/improbable)  Incentives  Punishment  Consequents  Reinforcement  Contingencies  Sanctions |
| Intentions | A conscious decision to perform a behaviour or a resolve to act in a certain way | Stability of intentions  Stages of change model  Transtheoretical model and stages of change |
| Goals | Mental representations of outcomes or end states that an individual wants to achieve | Goals (distal/proximal)  Goal priority  Goal/target setting  Goals (autonomous/controlled)  Action planning  Implementation intention |
| Memory, attention and decision processes | The ability to retain information, focus selectively on aspects of the environment and choose between two or more alternatives | Memory  Attention  Attention control  Decision making  Cognitive overload/tiredness |
| Environmental context and resources | Any circumstance of a person’s situation or environment that discourages or encourages the development of skills and abilities, independence, social competence and adaptive behaviour | Environmental stressors  Resources/material resources  Organisational culture/climate  Salient events/critical incidents  Person × environment interaction  Barriers and facilitators |
| Social influences | Those interpersonal processes that can cause individuals to change their thoughts, feelings, or behaviours | Social pressure  Social norms  Group conformity  Social comparisons  Group norms  Social support  Power  Intergroup conflict  Alienation  Group identity  Modelling |
| Emotion | A complex reaction pattern, involving experiential, behavioural, and physiological elements, by which the individual attempts to deal with a personally significant matter or event | Fear  Anxiety  Affect  Stress  Depression  Positive/negative affect  Burn-out |
| Behavioural regulation | Anything aimed at managing or changing objectively observed or measured actions | Self-monitoring  Breaking habit  Action planning |
