# Supplementary material for: Key influences on university students’ physical activity: a systematic review using the Theoretical Domains Framework and the COM-B model of human behaviour
Source: BMC Public Health. 2024 Feb 9;24:418. doi: 10.1186/s12889-023-17621-4 (PMC10854129; doi:10.1186/s12889-023-17621-4)
Supplement: Supplementary file 2 — Additional file 2. Search syntax for Ovid MEDLINE. [file 12889_2023_17621_MOESM2_ESM.docx]

Additional file 2. Search syntax for Ovid MEDLINE

1. exercis*.tw.

2. exp Exercise/

3. physical activit*.tw.

4. exp Physical Fitness/

5. exp Sports/

6. inactive.tw.

7. sedentary.tw.

8. 1 or 2 or 3 or 4 or 5 or 6 or 7

9. universit*.tw.

10. colleg*.tw.

11. undergrad*.tw.

12. baccalaureate.tw.

13. tertiary.tw.

14. 9 or 10 or 11 or 12 or 13

15. student*.tw.

16. exp Students/

17. 15 or 16

18. 14 and 17

19. intervention*.tw.

20. (program or programs or programme*).tw.

21. (campaign or campaigns).tw.

22. exp Health Promotion/

23. facilitator*.tw.

24. enabl*.tw.

25. motiv*.tw.

26. promot*.tw.

27. barrier*.tw.

28. inhibit*.tw.

29. (behavio?r chang*).tw.

30. 19 or 20 or 21 or 22 or 23 or 24 or 25 or 26 or 27 or 28 or 29

31. 8 and 18 and 30

32. limit 31 to (english language and yr="2010 -Current")
