# Supplementary material for: Key influences on university students’ physical activity: a systematic review using the Theoretical Domains Framework and the COM-B model of human behaviour
Source: BMC Public Health. 2024 Feb 9;24:418. doi: 10.1186/s12889-023-17621-4 (PMC10854129; doi:10.1186/s12889-023-17621-4)
Supplement: Supplementary file 3 — Additional file 3. Summary of study characteristics. [file 12889_2023_17621_MOESM3_ESM.docx]

| **Additional file 3.** Summary of study characteristics | | | | | | | |
| --- | --- | --- | --- | --- | --- | --- | --- |
| **Author** | **Country** | **Participants** | **Student enrolment status** | **Physical activity** | **Design** | **Method** | **Analytical approach** |
| Aljayyousi et al., 2019 | Qatar | N = 20  n female = 10 (50%)  n male = 10 (50%)   Mean age (SD) = N/A | Undergraduates | Mixed physically active and inactive | Qualitative | Semi-structured interviews | Inductive qualitative analyses |
| Awadalla et al., 2014 | Saudi Arabia | N = 1,257  n female = 831 (66.1%)  n male = 426 (33.9%)  Mean age (SD) = 20.1 (1.40) | N/A | Mixed physically active and inactive | Quantitative | Self-report survey/questionnaire | Response frequency |
| Bellows-Riecken, Mark & Rhodes, 2013 | Canada | N = 126  n female = 81 (70%)  n male = 45 (30%)  Mean age (SD) = 22.26 (6.04) | Undergraduates | Mixed physically active and inactive | Qualitative | Self-report survey/questionnaire | Content analysis |
| Brunette et al., 2011 | Canada | N = 14  n female = 5 (35.7%)  n male = 9 (64.3%)  Mean age (SD) = N/A | Undergraduates | Physically active | Qualitative | Semi-structured interview | Interpretative phenomenological analysis |
| Burton et al., 2021 | United Arab Emirates | N = 25  n female = 25 (100%)  n male = 0  Mean age (SD) = 20.4 (2.6) | N/A | N/A | Qualitative | Focus groups | Inductive thematic analysis |
| Chaabna et al., 2022 | Qatar | N = 370  n female = 229 (61.8%)  n male = 141 (38.2%)   Mean age (SD) = 20.1 (3.0) | Mixed undergraduates and postgraduates | Mixed physically active and inactive | Quantitative | Self-report survey/questionnaire | Response frequency |
| Cooke et al., 2015 | United Kingdom | N = 26 (13 control; 13 intervention)   n female = 81 (59.6%)  n male = 55 (40.4%)  Mean age (SD) = 22.2 | Undergraduates | N/A | Qualitative (post-intervention) | Focus groups | Thematic analysis |
| Deliens et al., 2015 | Belgium | N = 46  n female = 29 (63%)  n male = 17 (37%)  Mean age (SD) = 20.7 (1.6) | Undergraduates | Mixed physically active and inactive | Qualitative | Focus groups | Thematic analysis (inductive) |
| Devine, 2016 | USA | N = 16  n female = 9 (56.3%)  n male = 7 (43.7%)  Mean age (SD) = 20.56 (2.16) | Undergraduates | Mixed physically active and inactive | Qualitative | In-depth open-ended interviews | Grounded theory |
| Diehl et al., 2018 | Germany | *Quantitative* N = 689  n female = 479* (69.5%)  n male = 210* (30.5%)  Mean age (SD) = 22.69 (2.73)  *Qualitative* N = 20  n female = N/A  n male = N/A  Mean age (SD) = 22.8 (N/A) | Mixed undergraduates and postgraduates | Mixed physically active and inactive | Mixed-methods | *Quantitative* Self-report survey/questionnaire  *Qualitative* Semi-structured interviews | *Quantitative* Response frequency  *Qualitative* Content analysis |
| El-Gilany et al., 2011 | Egypt | N = 1,708  n female = 856 (50.1%)  n male = 852 (49.9%)  Mean age (SD) = 19.6 (1.6) | N/A | Mixed physically active and inactive | Quantitative | Self-report survey/questionnaire | Response frequency |
| Forrest & Bruner, 2017 (Phase II) | Canada | N = 23  n female = N/A  n male = N/A  Mean age (SD) = N/A | Undergraduates | N/A | Qualitative (post-intervention) | Focus groups | Thematic content analysis |
| Goldstein et al., 2017 | USA | N = 157  n female = 82 (52.2%)  n male = 75 (47.8%)  Mean age (SD) = 21.78 (5.17) | Undergraduates | Mixed physically active and inactive | Quantitative | Self-report survey/questionnaire | Response frequency |
| Griffiths et al., 2022 | United Kingdom | *Quantitative* N = 729  n female = 481 (66%)  n male = 248 (34%)  Mean age (SD) = N/A *Qualitative* N = 27  n female = N/A  n male = N/A  Mean age (SD) = N/A | Undergraduates | Mixed physically active and inactive | Mixed-methods | *Quantitative* Self-report survey/questionnaire  *Qualitative* Semi-structured interviews and focus groups | *Quantitative:* Response frequency   *Qualitative:* Thematic analysis |
| Hilger-kolb | Germany | *Quantitative* N = 689  n female = 479 (69.5%)  n male = 210 (30.5%)  Mean age (SD) = 22.7 (2.7)  *Qualitative* N = 20   n female = 13 (65%)  n male = 7 (35%)  Mean age (SD) = 22.8 (N/A) | Mixed undergraduates and postgraduates | Mixed physically active and inactive | Mixed-methods | *Quantitative* Self-report survey/questionnaire  *Qualitative* Semi-structured interviews | *Quantitative* Response frequency  *Qualitative* Content analysis |
| King et al., 2014 | USA | N = 480  n female = 318 (66.3%)  n male = 162 (33.8%)  Mean age (SD) = 19.68 (1.76) | Mixed undergraduates and postgraduates | Mixed physically active and inactive | Quantitative | Self-report survey/questionnaire | Response frequency |
| Kwan & Faulkner, 2011 | Canada | N = 45  n female = 26 (57.7%)  n male = 19 (42.3%)  Mean age (SD) off-campus students = 18.73 (.89)  Mean age (SD) residence students = 18.64 (0.98) | Undergraduates | Mixed physically active and inactive | Qualitative | Focus groups | Thematic analysis (deductive) |
| LaCaille et al., 2011 | USA | N = 49   n female = 32 (65.3%)  n male = 17 (34.7)  Mean age (SD) = 19.3 (1.2) | Undergraduates | N/A | Qualitative | Focus groups | Thematic analysis |
| Leinberger-Jabari et., 2023 | United Arab Emirates (Abu Dhabi) | N = 35  n female = N/A  n male = N/A  Mean age (N/A) = N/A | Undergraduates | N/A | Qualitative | Focus groups | Thematic analysis |
| Lerner, Burns & de Roiste, 2011 | Ireland | *Quantitative  N = 532* n female = 335 (63%)  n male = 197 (37%)  Mean age male (SD) = 21 (3.20)  Mean age female (SD) = 21.2 (4.2)  *Qualitative* N = 16  n female = N/A  n male = N/A  Mean age (SD) = N/A | Undergraduates | Mixed physically active and inactive | Mixed-methods | *Quantitative* Self-report survey/questionnaire  *Qualitative* Semi-structured interviews | *Quantitative* Response frequency  *Qualitative* Content analysis |
| Marmo, 2013 | USA | N = 56  n female = 31 (55.4%)  n male = 25 (44.6%)  Mean age (SD) = 23.55 | Undergraduates | Mixed physically active and inactive | Qualitative | Focus groups | Thematic analysis |
| Miyawaki et al., 2019 | Japan | N = 499  n female = 92 (18.4%)  n male = 407 (81.6%)  Mean age (SD) male non-exercise group = 19.1 (1.1)  Mean age (SD) male exercise group = 19 (1.0)  Mean age (SD) female non-exercise group = 18.8 (0.7)  Mean age (SD) female exercise group = 18.9 (0.6) | N/A | Mixed physically active and inactive | Quantitative | Self-report survey/questionnaire | Response frequency |
| Monforte et al., 2021 | Spain | N = 27  n female = 13 (48%)  n male = 14 (52%)  Mean age (SD) = 40.29 (12.22) | N/A | N/A | Qualitative | Interviews | Thematic analysis |
| Musaiger et al., 2014 | Kuwait | N = 530  n female = 327 (61.7%)  n male = 203 (38.3%)  Mean age male (SD) = 21.5 (3.5)  Mean age female (SD) = 20.6 (2.6) | N/A | N/A | Quantitative | Self-report survey/questionnaire | Response frequency |
| Nannyonjo et al., 2013 | Uganda | *Quantitative* N = 150  n female = 150 (100%)  n male = 0  Mean age (SD) = N/A  *Qualitative* N = 24  n female = 24 (100%)  n male = 0   Mean age (SD) = N/A | Undergraduates | Mixed physically active and inactive | Mixed-methods | *Quantitative* Self-report survey/questionnaire  *Qualitative* Focus groups | *Quantitative* Response frequency  *Qualitative* Thematic analysis |
| Nolan, Sandada & Surujlal, 2011 | South Africa | N = 462  n female = 268 (58%)  n male = 194 (42%)  Mean age (SD) = 19.98 (2.25) | Mixed undergraduates and postgraduates | Mixed physically active and inactive | Quantitative | Self-report survey/questionnaire | Response frequency |
| Othman et al., 2022 | Malaysia | N = 26  n female = 26 (100%)  n male = N/A   Mean age (SD) = 23 (1.17) | Undergraduates | Mixed physically active and inactive | Qualitative | Interviews | Inductive and deductive thematic analysis |
| Pan & Nigg, 2011 | USA | N = 32  n female = 14 (43.8%)  n male = 18 (56.2%)  Mean age (SD) = 25.3 (6.1) | N/A | N/A | Qualitative | Focus groups and semi-structured interview | N/A |
| Pellerine et al., 2022 | Canada | N = 341  n female = 279 (81%)  n male = 62 (19%)  Mean age (SD) = 23 (4) | Undergraduates | Mixed physically active and inactive | Quantitative | Self-report survey/questionnaire | Logistic regression model |
| Quintiliani et al., 2012 | USA | N = 14  n female = 6 (42.9%)  n male = 8 (57.1%)  Mean age (SD) = 25 (N/A) | Undergraduates | N/A | Qualitative | Semi-structured interview | Systematic team-based analysis approach |
| Ramírez-Vélez et al., 2015 | Columbia | N = 5,663  n female = 2,315 (40.9%)  n male = 3,348 (59.1%)   Mean age (SD) = 20.8 (2.8) | Mixed undergraduates and postgraduates | Mixed physically active and inactive | Quantitative | Self-report survey/questionnaire | Response frequency |
| Ranasinghe et al., 2016 | Sri Lanka | *Quantitative* N = 113  n female = 76 (67.3%)  n male = 37 (32.7%)  Mean age (SD) = 23.4 (1.0) *Qualitative* N = 87  n female = 51  n male = 36  Mean age (SD) = 22.8 (1.1) | Undergraduates | Mixed physically active and inactive | Mixed-methods | *Quantitative* Interviewer-administered questionnaire  *Qualitative* Focus groups and in-depth interviews | *Quantitative* Response frequency, Chi square and t-tests  *Qualitative* Directive content analysis |
| Romaguera et al., 2011 | Spain | N = 2,051  n female = 1,188 (57.9%)  n male = 863 (42.1%)  Mean age (SD) = 21.9 (4.8) | Mixed undergraduates and postgraduates | Mixed physically active and inactive | Quantitative | Self-report survey/questionnaire | Response frequency |
| Silver et al., 2019 | Canada | *Quantitative N = 477*  n female = 317 (66.5%)  n male = 160 (33.5%)  Mean age (SD) men = 19.9 (2.5)  Mean age (SD) women = 19.9 (2.0) *Qualitative* n = 41  n female = 16 (39%)  n male = 25 (61%)  Mean age (SD) women = N/A | Undergraduates | Mixed physically active and inactive | Mixed-methods | *Quantitative* Self-report survey/questionnaire  *Qualitative* Focus groups | *Quantitative* Logistic regression  *Qualitative* Thematic analysis |
| Snyder et al., 2017 | USA | N = 24  n female = 16 (66.7%)  n male = 8 (33.3%)  Mean age (SD) = 21.84 (1.06) | Undergraduates | Physically active | Mixed-methods | *Quantitative* Self-report survey/questionnaire  *Qualitative* Interviews | *Quantitative* Response frequency and MANOVA  *Qualitative* Interpretive qualitative research analysis |
| Tong et al., 2018 | Australia | N = 55  n female = 28 (51%)  n male = 27 (49%)  Mean age (SD) = 23.6 (4.6) | N/A | N/A | Qualitative (pre- and post- intervention) | Semi structured, in-depth interviews and focus groups | Thematic analysis (inductive) |
| von Sommoggy et al., 2020 | Germany | N = 46  n female = 28 (60.9%)  n male = 18 (39.1%)  Mean age students from university 1 (SD) = 23.6 (2.2)   Mean age students from university 2 (SD) = 23.8 (2.8) | N/A | Mixed physically active and inactive | Qualitative | Focus groups | Thematic analysis |
| Walsh, Taylor & Brennick, 2018 | Canada | N = 48  n female = 28 (58%)*  n male = 21 (43%)*  Mean age (SD) = N/A | N/A | N/A | Qualitative | Self-report audio-recording and focus groups | Thematic analysis |
| Wilson et al., 2019 | New Zealand | N = 121  n female = N/A  n male = N/A  Mean age (SD) = N/A | Undergraduates | N/A | Mixed-methods | Self-report survey/questionnaire | *Quantitative* Response frequency  *Qualitative* Thematic analysis (inductive) |
| *Errata originates from source paper; included for transparency | | | | | | | |
